# Research progress on iron absorption, transport, and molecular regulation strategy in plants

**DOI:** 10.3389/fpls.2023.1190768

**Published:** 2023-07-03

**Authors:** Xinyi Ning, Mengfei Lin, Guohua Huang, Jipeng Mao, Zhu Gao, Xiaoling Wang

**Affiliations:** ^1^ Institute of Biological Resources, Jiangxi Academy of Sciences, Nanchang, China; ^2^ College of Environmental And Chemical Engineering, Nanchang Hangkong University, Nanchang, China; ^3^ Kiwifruit Engineering Research Center of Jiangxi Province, Nanchang, China; ^4^ JInstitute of Biotechnology, Jiangxi Academy of Sciences, Ji’an, Jiangxi, China

**Keywords:** plant, iron, absorption, transport, molecular regulation

## Abstract

Iron is a trace element essential for normal plant life activities and is involved in various metabolic pathways such as chlorophyll synthesis, photosynthesis, and respiration. Although iron is highly abundant in the earth’s crust, the amount that can be absorbed and utilized by plants is very low. Therefore, plants have developed a series of systems for absorption, transport, and utilization in the course of long-term evolution. This review focuses on the findings of current studies of the Fe^2+^ absorption mechanism I, Fe^3+^ chelate absorption mechanism II and plant-microbial interaction iron absorption mechanism, particularly effective measures for artificially regulating plant iron absorption and transportation to promote plant growth and development. According to the available literature, the beneficial effects of using microbial fertilizers as iron fertilizers are promising but further evidence of the interaction mechanism between microorganisms and plants is required.

## 1 Introduction

Iron is the most abundant essential trace element in human body (Obi et al., 2022). Fe deficiency is considered to be the first of the three “hidden hunger” in the world, with approximately one in five people worldwide suffering from iron deficiencyanemia (Lowe N.M., 2021). As the fundamental food source for animals and human beings, plants can directly absorb iron nutrition from the soil environment and are direct carriers of iron received from the soil (Krishna et al., 2023). Iron is the first identified essential plant element that plays an important role in the whole plant growth cycle, and it is involved in the process of life activities such as protein synthesis, DNA replication, and respiration in the plant (Takanori et al., 2018), as well as in the composition of chlorophyll and plays an important role in biological nitrogen fixation. Nearly 40% of arable plants worldwide show varying degrees of iron deficiency yellowing, especially in calcareous soils in arid and semi-arid regions, where iron deficiency in plants is widespread. Worldwide, the North American continent, the Mediterranean coast, and parts of South America are severely iron deficient. In China, iron deficiency occurs in areas from Sichuan Basin in the south to the Inner Mongolian Plateau in the north, Huaibei Plain in the east to the Loess Plateau in the west, Gansu, Qinghai, Xinjiang and other provinces (Satoshi, 1999). In general, Fe exists in the soil mainly in two valence states, Fe2+ and Fe3+, of which Fe2+ can be absorbed and utilized by plants. While Fe_3+_ is difficult to be absorbed by plant roots due to its low solubility and poor effectiveness. The active Fe content in soil is easily affected by factors such as soil pH, salts, and some ions. For example, under acidic and flooded conditions, it exists as dissolved Fe_2+_; when the soil pH is greater than 6.5, the content of active Fe starts to decline and exists in an insoluble or insoluble state; when the pH exceeds 8.5, it exists mainly in insoluble iron carbonate form, causing the appearance of yellowing in plants (Huei-hsuan and Wolfgang, 2020). Iron in alkaline soils often combines with phosphate or hydroxyl ions to form precipitates, resulting in the inability to be absorbed by plants (Fu et al., 2017). Plants have evolved a series of molecular systems for Fe uptake and translocation both to obtain sufficient Fe from the soil and to avoid Fe toxicity due to excessive uptake. Depending on the uptake mechanism, they can be divided into mechanism I and mechanism II. Mechanism I mainly absorb Fe_2+_, which consists of three systems: the H+-ATPase pump system, the Fe_3+_ reduction system, and the translocation system of Fe2+, which are in turn influenced by ILR3 (Tissot et al., 2019), YABBY (Sun et al., 2021), the FEP1, IMA3 (Okada et al., 2022), MNB1 (Song et al., 2022), and bHLH1b (Liang, 2022); Mechanism II is achieved through Fe3+ chelate uptake, which is synthesized by the lysergic acid-like plant iron carrier (phytosiderophore, PS). The genes associated with this mechanism are GRX (Kobayashi et al., 2022a), ACOs (Senoura et al., 2020), HRZ (Liang, 2022) and so on. There is also another reported mechanism: the mechanism of interaction between microorganisms and plants. This mechanism is regulated by genes such as NRAMP (Cailliatte et al., 2010) and TonB (Bruce et al., 2021), and plants can take up iron by interacting with microorganisms. Since the iron is difficult to be absorbed and utilized by plants in the soil environment, plants are facing the problem of iron deficiency stress. To explore ways to solve this difficult problem, this paper provides a comprehensive elucidation of the adaptive and non-adaptive mechanisms of Fe uptake by plants from plant morphology, physiological and biochemical, molecular regulatory mechanisms, and metabolic pathways and analyzes the regulatory networks and mechanisms of action associated with each type of mechanism, proposing insights into the study of Fe uptake and utilization by plants, to provide references for carrying out Fe recycling and utilization in the plant body.

### 1.1 Effect of iron on plant morphology and physiology

#### 1.1.1 Effect of iron on plant morphological characters

Iron is not a constituent of chlorophyll, but it is involved in the process of chlorophyll synthesis (Norman and Javier, 1986). When plants are deficient in iron, they first show symptoms such as vein chlorosis, shrunken leaves, and plant dwarfing, which in turn affect the normal growth and development of the plant, leading to damage to fruit quality, serious yield loss of cash crops, even worse, it can cause the entire plant to die. The symptoms of iron deficiency in plants vary from species to species. In mild iron deficiency, young leaves lose green between veins, while the veins remain green, and the leaves become smaller and thinner; in severe iron deficiency, the veins also begin to fade to green, stem growth is hindered, and the terminal buds wither; in more severe cases, the leaves all lose green or even turn white, the leaf flesh is necrotic, the leaf edges turn red, the leaf tips and leaf edges are necrotic and scorched, and the stems begin to stop growing, such as apple and peach (Sun et al., 1987; Victoria et al., 2008); it also causes leaf loss of green color, and the length of new internodes, leaf weight, and leaf thickness is significantly reduced, such as grapes and pears (Victoria et al., 2008; José et al., 2016).

The root system, as the main organ for nutrient uptake by plants, adapts to iron deficiency through corresponding changes in root morphology and physiology when the iron content in the soil is reduced (Maribela et al., 2012). Iron deficiency causes plants to appear enlarged and thickened near the root tip, with an increase in root hairs, and to cope with iron deficiency adversity by secreting large amounts of H^+^ and enhancing the activity of trivalent ferric ion chelating reductase, thereby increasing the effectiveness of iron in plant roots, as in cucumber and tomato; it also results in rapid growth and thickening of the root tip and the continuous accumulation of more and more phenolic compounds in the epidermis and cortex, as a way to reduce insoluble ferric ion compounds, thus slowing down the damage caused by Fe deficiency, such as soybean (Yi et al., 1998); it also leads to main root growth, reduced root number, increased root hairs, increased root dry weight, and increased root surface area, such as wheat and pear (Fan et al., 2018; Zeng et al., 2019).

#### 1.1.2 Effect of iron on plant physiology

##### 1.1.2.1 Chlorophyll synthesis

The chloroplasts in most plant leaves contain about 80% of iron (Giovanni et al., 2015), of which 60% is immobilized in the cystoid membrane, 20% in the chloroplast stroma, and only a very small fraction of iron is in other organs. Therefore, the impeded chlorophyll synthesis due to iron deficiency is the main cause of leaf greening and yellowing.

In iron-deficient environments, ultrastructural abnormalities were observed in plant chloroplasts and chloroplasts, and the number of cystoid membranes in the lamellar structure of plant chloroplasts was reduced (Zhou et al., 2008). Iron deficiency led to a decrease in cis-aconitase activity, which is inextricably linked to the synthesis of chlorophyll precursors. Iron deficiency chlorophyll synthesis, chlorophyll content, and concentration in leaves decrease with increasing iron deficiency yellowing, as in balsam pear (Zeng et al., 2019) and kiwifruit (Wang et al., 2019); iron deficiency leads to a significant reduction of chloroplast basidia and stromal cysts in faded green leaves, with disrupted ultrastructure and significantly reduced chlorophyll concentration, common in iron deficiency-sensitive tangerine (Ding et al., 2016); More seriously, not only the leaf chloroplast structure is severely affected, but also the mesophyll cells, palisade cells and parenchyma cells of the main vein (Zhang et al., 2019), as in Mexican Lime (Ranferi et al., 2006).

##### 1.1.2.2 Photosynthesis

PS-I (photosystem-I) is the photosynthetic apparatus with the highest Fe content in the entire electron transport chain, and studies in a variety of plants have shown that Fe deficiency leads to severe disruption of the structure and activity of PS-I. Current studies have reported that plant photosynthesis is affected by Fe in three ways: first of all, Fe affects the production of cystoid in plant chloroplasts, and Fe deficiency stress disrupts the formation of photosynthetic elements and has a direct effect on photosynthetic basic substances and sites of action (Yoshida et al., 2021); afterwards, many Fe-oxygen complexes intervene in the process of photochemical reactions in plants, such as cytochrome C oxidase complex, Fe oxygen reductase, heme, and bean heme, and in Fe deficiency adversity, the proportion of the content of these substances shrinks and the physiological activities of some Fe-related enzymes are abrogated (Agarwala et al., 1965; Yadavalli et al., 2012); finally, Fe is an influential factor in the electron transfer process in photosynthesis, and in Fe deficiency, interrupts the electron transfer chain and hinders the photolysis of water, thus blocking the process of photosynthesis, and can this leads to a decrease in the capacity of ROS (reactive oxygen species) detoxification enzymes, which ultimately leads to a negative impact on the photosynthetic rate of plants (Singh et al., 2005).

The factors that lead to the decrease of photosynthetic rate include stomatal limitation and non-stomatal limitation. Stomatal limitation means that the weakening of photosynthesis is caused by the diffusion of CO_2_ to the carboxylation site caused by the decrease of stomatal opening, rather than the stomatal limitation that makes the CO_2_ concentration in the intercellular space very high, but photosynthesis is still limited very weakly. This can be judged by the magnitude and trend of Ci (Intercellular CO_2_ concentration) values, where poplar exhibits higher stomatal restriction with higher stomatal conductance and more CO_2_ lost by photosynthesis (Jurca et al., 2022). In iron-deficient chlorosis ‘Dangshan crisp pear’, the Gs (stomatal conductance) of leaves decreased and Ci increased, which means that the decrease of Pn (Net-photosynthetic-rate) accompanied by the increase of Ci, and the decrease of the photosynthetic rate due to yellowing belonged to non-stomatal limitation.

##### 1.1.2.3 Respiration

Iron is associated with the composition of several respiration-related enzymes and is one of the basic components of these enzymes. And such enzymes are involved in plant respiration, such as cytochrome, CAT (catalase), and POD (peroxidase). Iron deficiency weakens the activity of these enzymes, causing a sudden decrease in the effectiveness of a series of redox reactions in plants, hindering the normal electron transfer and reducing ATP synthesis, thus making respiration affected (Sandmann, 1985). Iron is a cofactor of cis-aconitase, the rate-limiting enzyme of the entire respiration of the TCA cycle, and when plants are in an iron-deficient environment, the activity of this enzyme is inhibited and the process of regulating respiration is affected. Electron transport also requires the assistance of iron-sulfur proteins, and the complexes in the respiratory electron transport chain process all contain iron-sulfur proteins, and iron deficiency interferes with the activity of these protein complexes, further affecting the rate of transport of the respiratory electron transport chain in plants and ultimately hindering plant growth and metabolism (Philip and Leonid, 2005).

##### 1.1.2.4 Metabolism of nitrogen element and other products

Iron plays a non-negligible role in the microbial nitrogen cycle, it is involved in the metabolic processes of nitrogen and is an important component of some essential enzymes in nitrogen fixation reactions, such as nitrogen-fixing enzymes. In the presence of iron deficiency, nitrate reductase activity is reduced, while glutamine synthetase and glutamate synthetase levels are increased (Borlotti et al., 2012). Apples supply nitrate for uptake by using nitrate reductase and nitrite reductase to reduce nitrate to nitrite, which is further reduced to ammonia and finally synthesizes amino acids, proteins, and other substances (Sun et al., 2021b). It has also been shown that nitrogen nutritional status is a key determinant of iron reactivation in plants and regulates the transfer of iron from fully developed senescing leaves wanting to grow sites (Shamima et al., 2018).

Iron deficiency also causes the development of plant diseases, reduced quality, and lower yields. It has been found that iron inhibits the growth of tomato cyanobacteria and alleviates disease development (Truchon et al., 2022). Iron affects fruit quality and yield by influencing plant enzyme activity and metabolism. In Fe-deficiency, apple induces increased enzyme activities of POD, superoxide SOD (dismutase), and root iron ion chelating reductase (FRO) under adversity (Li, 2003). Espen et al. (2000) used 31P-NMR to analyze the metabolic responses of cucumber roots under Fe-deficiency conditions and found that Fe-deficiency induced activation of metabolism and depletion of stored carbohydrates. Iron deficiency significantly reduced the iron content in citrus rootstocks and the sugar content in leaves (Mary-Rus et al., 2013); strawberry fruit quality and yield were affected, and in severe cases plants were unable to hang fruit (Zhang, 2020); the application of organic fertilizers containing iron too early for grapefruit increased the content of reducing sugars, total sugars, soluble solids, and vitamin C in the fruit.

## 2 Mechanism of iron uptake and transport in plants

The transport process of trace elements such as iron in plants first reaches the extra plastic body of root tip cells by diffusion, then the absorbed iron is transported from the extracellular to the intracellular by the iron carrier on the cell membrane, into the cellular cytoplasm, then into the xylem thin-walled tissue in the root system, during which divalent iron is oxidized to trivalent iron into the ducts of the roots, transported through the ducts to the leaf flesh cells of mature leaves, and then it is then redistributed in the leaves and finally reaches the growth cells through the bast. The main systems for long-distance iron transport in plants are the xylem and the phloem.

The transport of iron ions in the plant body: Root apoplast → root plastid → thin-walled cell tissue → ducts → leaf flesh cells → bast → developmental cells (Qu, 2005).

However, in the vast majority of soil environments, iron levels are often not sufficient for normal plant growth. Higher plant inter-roots have continuously evolved a series of mechanisms of iron deficiency stress tolerance to adapt to iron deficiency adversity. For example, adaptive mechanisms regulated by iron nutrition in plants, including Mechanism I and Mechanism II; mechanisms of plant-microbial interactions for iron uptake and transport.

### 2.1 Mechanism I of absorption by Fe^2+^

**Figure 1:**
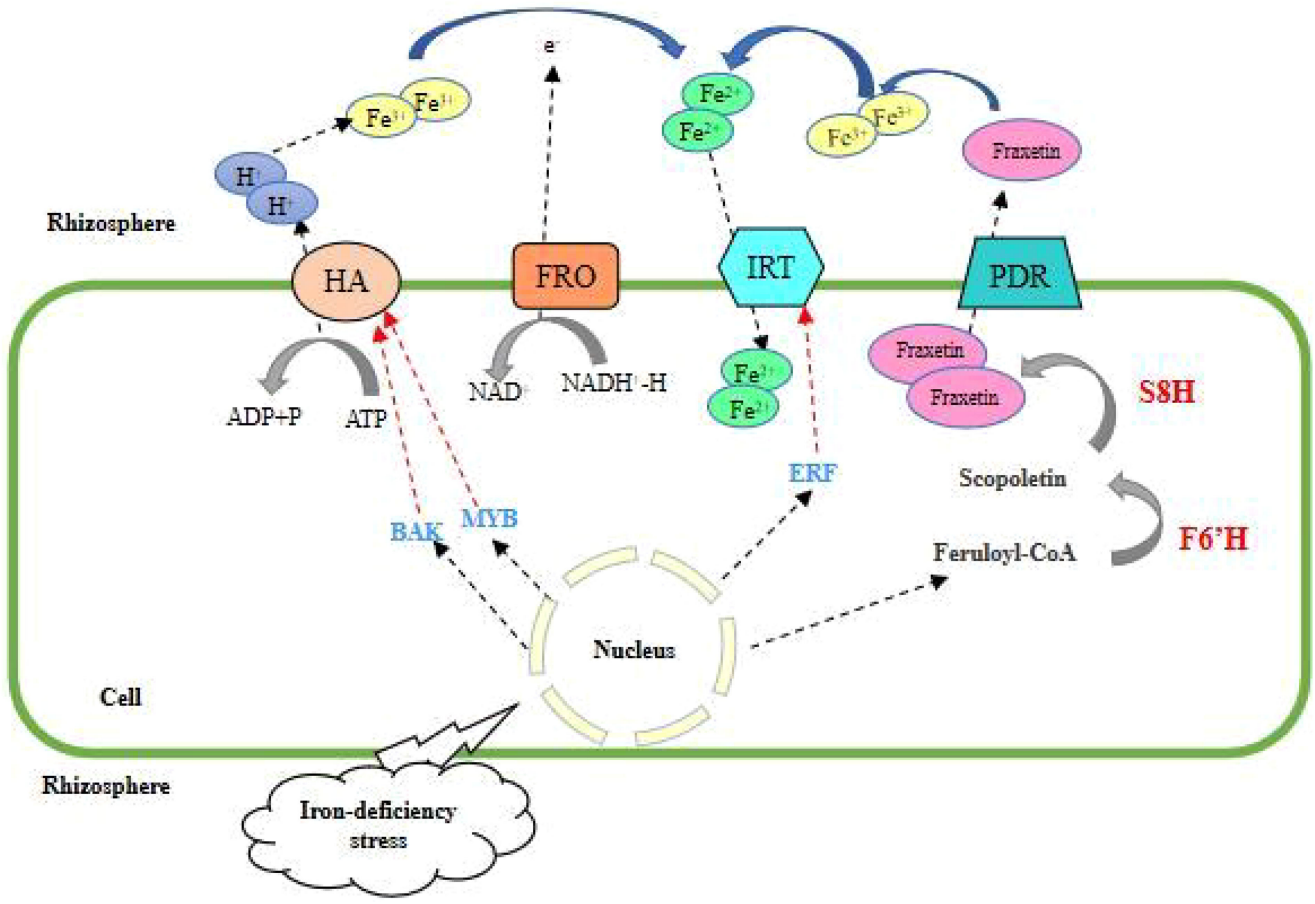
Mechanism I of plant root uptake and response to Fe deficiency stress.

The Fe uptake in mechanism I is accomplished by three protease systems: 1) H^+^-ATPase protease system, plant roots reduce the pH value of the surrounding soil by releasing hydrogen ions, and increase the dissolution of iron in rhizosphere soil. With the enhancement of H^+^-ATPase activity, the roots secrete coumarin and riboflavin to increase the mobility of Fe^3+^(Robe et al., 2020a; Robe et al., 2020b). Among the numerous H^+^-ATPase (HA) genes, the expression of some HA genes is induced by iron deficiency stress, among which AHA2 is a direct commitment protein for hydrogen ion secretion, and ABA-activated BAK1 phosphorylates AHA2 at its C-terminal Ser-944 and activates AHA2, leading to rapid hydrogen ion efflux, cytoplasmic alkalinization, and ROS accumulation (Pei et al., 2022), and *CsHA1* in cucumber plays a similar role (Santi et al., 2003). Meanwhile, the plasma membrane-localized H^+^-ATPase HA6 regulates hydrogen ion efflux and the expression of this gene is expressed up-regulated in plants grown on soils with low Fe content, while MYB308 activates *HA6* to promote inter-root hydrogen ion efflux and Fe uptake (Fan et al., 2022). 2) Fe^3+^ reduction system, consisting of trivalent iron chelating reductase (FRO) and reductive coenzyme II (NADPH) dehydrogenase. Fe^3+^ released by the H^+^-ATPase protease system enters the cell after forming chelates with chelators in the plant, and the reduction system converts Fe^3+^ chelates into Fe^2+^ chelates released for plant uptake and utilization. The iron deficiency-induced pea Fe^3+^ reductase gene, *PsFRO1*, was expressed in plant roots, mycorrhizae, stems, and leaves, and the expression was relatively higher in root epidermal cells with Fe^3+^ reduction system and Fe^2+^ transporter protein system; and the expression of *PsFRO1* in the nitrogen fixation zone of root nodules may play a role in plant nitrogen fixation (Waters et al., 2002); natural allelic variation of *FRO2* regulates *Arabidopsis* root growth under iron deficiency conditions (Satbhai et al., 2017). This step is particularly important as it is the limiting step in the mechanism I process. 3) Fe^2+^ transporter protein (IRT) system, which brings Fe^2+^ chelates reduced by Fe^3+^ chelating reductase into the cell by transmembrane transport and then transported by other transporter proteins to various organelles and organs of the plant to supply plant growth and development. The *Arabidopsis AtIRT1* gene was the first *IRT* gene obtained by heterologous expression in a transporter double mutant yeast that eventually complemented iron deficiency symptoms (Eide et al., 1996). AtERF4 and AtERF72 are both negative regulators of the iron deficiency response and maintain iron homeostasis by directly binding to the *IRT1* promoter (Liu et al., 2017), AtERF4 through yeast single hetero ERF95 transcription factor regulates iron accumulation in *Arabidopsis* seeds through an *EIN3*-ERF95-*FER1*-dependent signaling pathway (Sun et al., 2020); ERF96 loses its function under iron deficiency stress and then increases the expression of iron uptake genes through ethylene and growth hormone signaling pathways and reduced expression of chlorophyll-degrading genes and enhanced iron and chlorophyll accumulation in *Arabidopsis* (Yao et al., 2022); NRANP is involved in the uptake of small amounts of iron from the soil by relying on its specific affinity for iron and its response to environmental signals (Eide et al., 1996); and INO reduces iron accumulation in developing seeds by inhibiting *NRANP1* expression to reduce iron loading in developing seeds. Phenolic compounds in *Arabidopsis* root secretions were also found to convert insoluble iron in the plant growth medium into soluble iron, but ultimately only absorbed from it through the IRT1/FRO2 high-affinity iron transport system (Vert et al., 2021). f6′H1 (Feruloyl coenzyme A 60-hydroxylase 1) is a key enzyme for Fe-deficiency-induced coumarin synthesis. In iron deficient environments, the biosynthesis of coumarin is of great significance for plant growth, promoting the absorption of iron and inducing plant immunity. Iron deficiency induces up-regulation of F6′H1 expression and shifts the lignin synthesis pathway to coumarin synthesis. The F6′H1 homolog S8H (scopoletin 8-hydroxylase), also induced by iron deficiency, was found to generate Fe^3+^-reducing fraxetin (7,8-dihydroxy-6-methoxy coumarin) by hydroxylating coumarin scopoletin, which can dissolve and bind iron to form stable complexes at neutral to alkaline pH (Zhang et al., 2021) (**Figure 1**) (Rajniak et al., 2018). Other genes such as *ILR3* (Tissot et al., 2019), *YABBY* (Sun et al., 2021a), *FEP1*, *IMA3* (Okada et al., 2022), *MNB1* (Song et al., 2022), and *bHLH1b* (Liang, 2022) are involved in this mechanism.

Soil pH and ${\text{HCO}}_{3}{\;}^{‐}$ have a strong influence on this mechanism, and bicarbonate stress induces plant roots to secrete relevant substances or protons into the inter-root soil to acidify it and inducing root iron reductase gene and iron transporter gene expression thereby enhancing iron reductase activity and iron uptake (Wang, et al., 2022). Plants that conform to the mechanism I for iron uptake mainly contain dicotyledons and non-grass monocotyledons, such as soybean, peanut, sunflower, and cucumber.

This strategy involves a protein complex consisting of HA, FRO, IRT and PDR. HA secreted protons to acidify the rhizosphere soil and mobilize Fe^3+^, and BAK and MYB assisted in the activation of HA. After being reduced by FRO, Fe^2+^ enters the plant roots with the help of IRT, and ERF negatively feeds back to regulate IRT. Coumarin also contributes to the reduction strategy, possibly by converting Fe^3+^ to Fe^2+^. Sideretin is the major coumarin at acidic pH, while fraxetin is the major coumarin at neutral or alkaline pH. PRD mediates coumarin efflux from roots.

### 2.2 Mechanism II of absorption as Fe^3+^ chelate

In plants, Fe must be bound to other compounds for transport, and substances such as citric acid, phenolics, NA (nicotinamide), and MAs (myristic acid) are involved in the transport of Fe in plants. FDR3 (citrate transporter protein) and OsFDRL1 in rice are involved in the transport of Fe by citrate loading in the xylem; NA and DMA (2’-deoxymyristic acid) are present in the secretions of the best and are involved in iron transport in the bast (Takanori et al., 2018). YSL has its presence in all parts of the plant, OsYSL15 is a Fe(III)-DMA transporter protein located on the plasma membrane and is mainly responsible for iron uptake from the inter-root (Inoue et al., 2009). OsYSL2 is a Fe(II)-NA transporter protein responsible for Fe transport in the bast (Ishimaru et al., 2010); OsYSL16 is present in the plasma membrane of periplasmic cells and is involved in Fe partitioning through the vascular bundle (Kakei et al., 2012); OsYSL18 is involved in Fe transport in reproductive organs and critical bast (Aoyama et al., 2009). OsYSL18 is related to Fe transport in reproductive organs and critical sclerotia (Aoyama et al., 2009).

**Figure 2:**
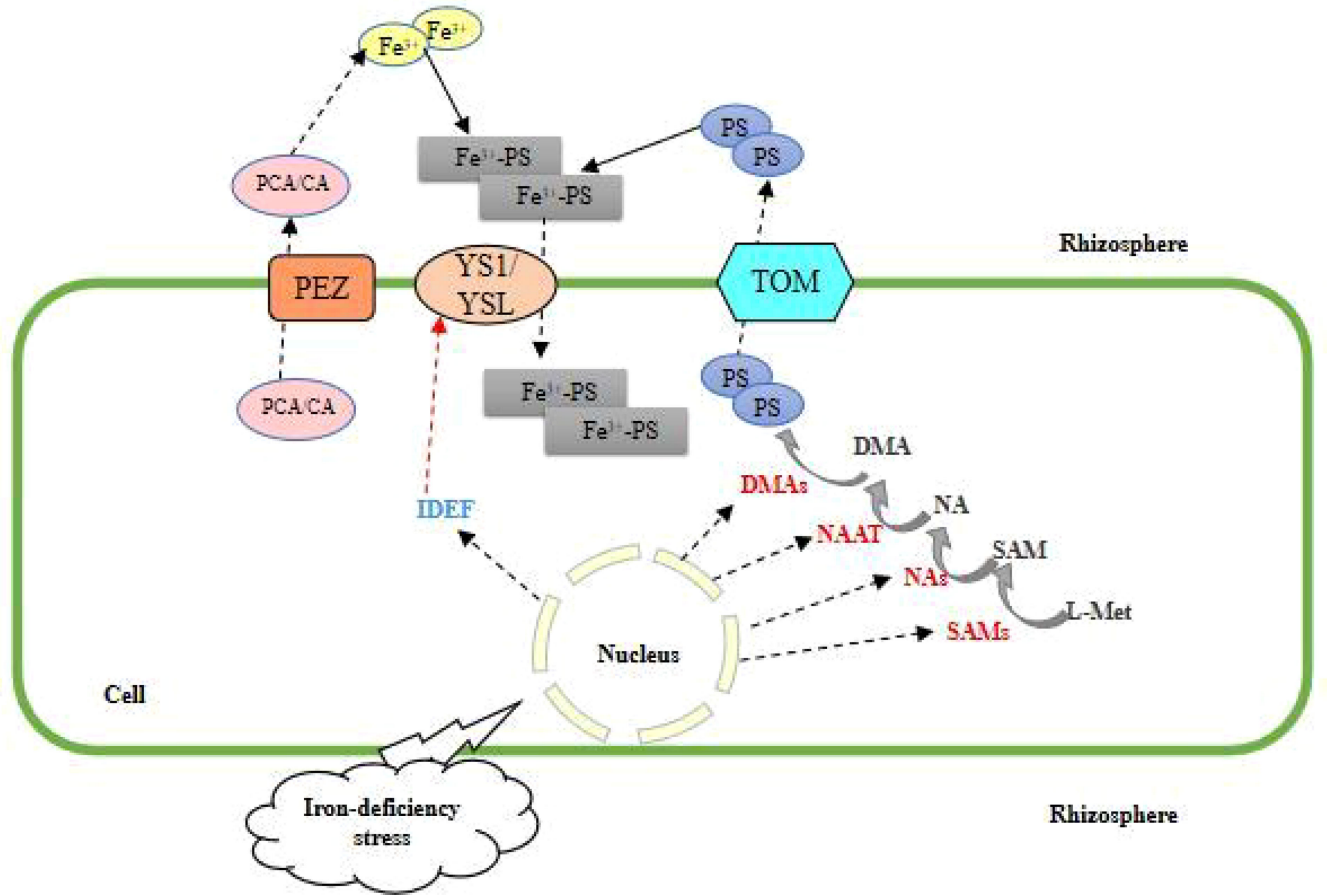
Mechanism II of plant root uptake and response to Fe deficiency stress.

MAs are a class of low molecular weight non-protein amino acids that have a strong affinity for trivalent Fe (with six functional groups that chelate Fe) and can form stable, octahedral trivalent chelates (Fe (III)-MAs) that are then taken up by plants. The trivalent chelates (Fe (III)-MAs) are stable and octahedral, which are then absorbed and used by plants. The adaptation of plants to Fe-deficient environments through mechanism II uptake occurs in two major steps: 1) the plant root system synthesizes the myristica acid-like PS (phytosiderophore) *via* SAM (S-adenosyl methionine) through a series of enzymatic reactions and then actively secretes it into the root environment *via* carrier action (Higuchi et al., 1999); 2) The phytosiderophore chelates with trivalent iron ions in the inter-root environment, forming Fe(III)-PS, a chelate of phytosiderophore and trivalent iron ions, and migrates to the root plasma membrane, where it is transported to the plant by the specific transporter proteins YS1 (yellow stripe 1), YSL (yellow stripe-like) and so on (Takahashi et al., 2012; Yamagata et al., 2022). Nicotinamide (NA), 2’-deoxycholic acid (DMA) and maltogenic acid (MA) are chelating agents required for the uptake and transport of iron by plants. While nicotinic amide synthetase (NAS), nicotinic amide aminotransferase (NAAT), 2’-deoxygenate synthase (double deoxygenate synthetase (DMAs), MAs transporter protein (TOM), and NA efflux transporter protein (ENA) are involved in iron uptake and transport in mechanism II plants (**Figure 2**). To adapt to the iron-deficient environment, DMA accumulates in the xylem sap of rice, the release of NA and DMA/MAs is regulated by different efflux transport proteins, and the expression of DMA efflux transport proteins OsTOM1, OsTOM2, and OsTOM3 is induced in roots, OsTOM1 is involved in the secretion of MAs to the root interiors, while OsTOM2 and OsTOM3 in some tissues exhibited specific expression patterns out of iron transport-related (Nozoye et al., 2015). Meanwhile, expression of the homolog of OsTOM1, vesicular creatine transporter (OsVMT), was induced in roots (Che et al., 2019). The histone or heme-associated protein (HAP) transcription factor (TF) HAP5A is required for the response to iron deficiency in *Arabidopsis*, and the expression of plant genes encoding nicotinamide synthase is greatly reduced in the presence of hap5a mutations (Xiao et al., 2020). Transfer of genes that are capable of efficient synthesis and secretion of myristic acid-like iron carriers in barley, such as *HvNAS*, *HvNAAT*, and *HvIDS3*, into rice significantly improved rice myristic acid synthesis and secretion (Motofumi et al., 2008); combination of carbohydrate-binding module family 11 (CBM11) and iron-binding peptide (IBP) into a CBM-IBP fusion peptide, secreted into the cell walls of *Arabidopsis* and rice, transformed *Arabidopsis* and rice plants showed significantly increased iron accumulation and biotransformation (Yang et al., 2016); based on the natural phycocyanin 2’-deoxymalginate (DMA), a novel iron chelator proline-2’- deoxymalginate (PDMA) solubilizes insoluble iron and upregulates the expression of the yellow streak family gene *AhYSL1* to improve iron nutrition in peanut plants (Wang et al., 2022). Other genes related to this mechanism include *ACOs* (Senoura et al., 2020) and *HRZ* (Liang, 2022). It has been shown that the combined introduction of *OsHRZ* knockdown and *OsIRT1* promoter-Refre1/372 can further improve iron deficiency tolerance without affecting the knockdown effect on iron accumulation (Kobayashi et al., 2022b).

Plants that take up iron by mechanism II are mainly monocotyledons, such as rice (*Oryza sativa* L.), wheat (*Triticum aestivum* L.), and maize (*Zea mays* L.). Plants have two distinctive features when they take up iron by mechanism II: carrier release and high affinity of iron carriers on root cell membranes. Soil pH had little effect on this mechanistic physiological process, so mechanism II plants in medium/alkaline soils showed no significant iron deficiency symptoms.

As a typical monocotyledonous plant, rice is not a typical mechanism II plant, which not only synthesizes DMA in the roots to chelate Fe^3+^ but also acquires Fe^2+^ through the transporter proteins OsIRT1 and OsIRT2. Three consecutive enzymatic reactions catalyzed by NAS, NAAT, and DMAS are also present in the synthesis of DMA. In addition, various transcription factors such as IDEF1, IDEF2, and bHLH were shown to regulate iron uptake and transport (Li et al., 2019).

In this mechanism, PEZ is responsible for secreting PCA/CA, reducing Fe^3+^to Fe^2+^, DMA excretes PS through TOM to chelate Fe^3+^, and Fe^3+^- PS is transferred to the root through YS1/YSL.

### 2.3 Microbial mechanism of plant iron uptake

**Figure 3:**
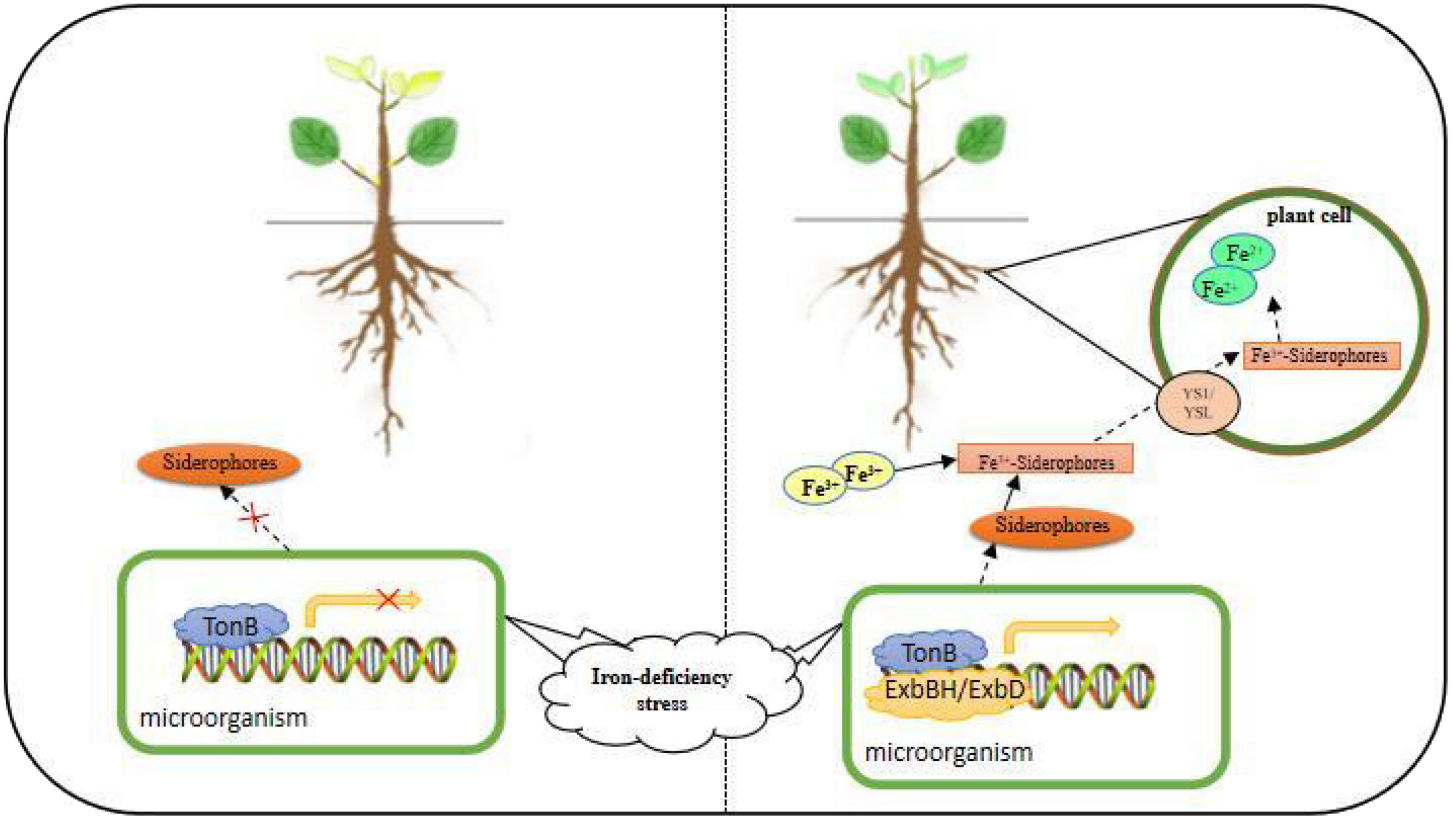
Microbial mechanisms of plant root uptake and response to Fe deficiency stress.

The mechanism of iron uptake and transport by plants cannot be fully explained by Mechanism I and Mechanism II alone. As an indispensable nutrient element for living organisms, the mechanism of its regulation in different organisms varies greatly. When the fungus-iron ion-plant triad interacts, it not only helps to enhance a new understanding of plant and microbial life activities but also facilitates a breakthrough in the field of plant disease resistance mechanism research (Qiu and Liu, 2022). Iron plays a role in plant-pathogen protection of plant host cells from bacterial or fungal infection. In recent years, it has been found that under low Fe conditions, microorganisms produce Fe carriers (Siderophores), and microbial Fe carriers can provide Fe nutrition to plants for their growth and development. There are two possible mechanisms for plant-microbe interactions: 1) microbial Fe carriers with high redox potential can be reduced to provide Fe(II) to the plant transport system (Konrad, 1994); 2) Microorganisms synthesized siderophores and diffused around the rhizosphere after iron deficiency stress, and siderophores chelated with Fe^3+^ to form Fe^3+^-Siderophores chelate. Part of the Fe^3+^-Siderophores chelate is absorbed by the microorganism itself and reduced to Fe^2+^ after entering the cell, which is used for the growth and development of the microorganism; the other part is absorbed and utilized by the plant (Gu et al., 2020). As a transcriptional repressor of iron carrier synthesis, Fur is the key factor, in dimensioning cellular iron homeostasis. When iron is abundant, Fur binds divalent iron to form a complex that binds to the promoters of iron carrier synthesis genes or regulatory genes to block their transcription; when cells are exposed to iron deficiency, Fur derepresses and induces iron carrier synthesis (Hosni and Bryan, 2013). The expression of the *TonB* gene depends on the availability of surrounding iron and the orientation from a specific gene *fur*, and expressed *TonB* is associated with *ExbBH* and *ExbD* interact to form TonB complexes that act through conformational changes to modulate outer membrane receptors, which in turn activate iron transport across the membrane (**Figure 3**) (Bruce et al., 2021). The product of the *NRAMP* gene helps prevent the synthesis of active defense enzymes by phagocytosed microorganisms by extracting metals from the phagocytic vesicles of the phagocytosed microorganisms, which contain iron or other metals as cofactors. In such mechanisms, ferritin attached to the surface of the plasma membrane may be invaginated by phagocytosis, and iron is digested by proteases secreted by the endoplasmic reticulum around the endocytic vesicles and separated from the protein, which then absorbs the free iron into the cytoplasm *via* the NRAMP transporter (Satoshi, 1999). In soybean plants, iron deficiency causes abnormal regulation of the *NRAMP* gene, and *GmNRAMP* expression was found in soybean root nodules and showed that this gene is capable of translocating iron or other metal ions in soybean root nodules (Qin et al., 2017). Maize and sunflower grew better in a non-sterile environment than in a sterile environment and had severe iron deficiency under sterile conditions (Masalha et al., 2000). The endophytic *Streptomyces* sp. GMKU3100 produced iron carriers in Thai rice and promoted rice and mung bean growth and development (Rungin et al., 2012). In Fe-deficient leaves, fungal infection progressed rapidly through biotrophic growth to necrotrophic growth development, whereas adequate Fe-nutrient status inhibited the formation of gram-derived anthracnose structures at the early biotrophic growth stage (Ye et al., 2014).

In this mechanism, the expressed TonB interacts with ExbBH and ExbD to form a TonB complex that acts through conformational change, modulates the outer membrane receptor, secretes ferriferous carrier, chelates Fe^3+^ in the environment, enters the plant root cell *via* YS1/YSL, and is reduced to Fe^2+^.

## 3 Techniques and methods for correcting iron deficiency in plants

### 3.1 Application of plant iron fertilizer

The application of iron fertilizer is the most direct and easy way to address the symptoms produced by iron deficiency in plants. Such as foliar spraying, soil application, and trunk injection. Leaf spraying mainly involves spraying yellowing leaves with iron salt solutions (iron citrate, EDHA-Fe (ethylene diamine tetraacetic acid-Fe), ferrous sulfate and so on), which can improve the yellowing disorder of plants, such as kiwifruit under the treatment of iron citrate and iron amino acid complex, which can effectively improve the chlorophyll content and the content of vitamin C, soluble solids and whole iron in fruits and improve fruit quality (Wang et al., 2011); the foliar spray of ferric citrate balanced the sugar-acid ratio of grape berries and increased the contents of anthocyanins, flavanols, and flavonols in grape skins (Zhang et al., 2022); after the application of iron fertilizer complex containing iron citrate Fe and EDDHA-Fe, it effectively increased the content of active Fe in peanuts at the flowering hypocotyl and ripening stages and also significantly increased the content of whole Fe in leaves and kernels (Liu et al., 2016); in using different combinations of ferrous sulfate with amino acids (AA), urea (Urea), and EDTA-2Na (disodium ethylene diamine tetraacetic acid) chelated Fe for yellowing apple fruit trees. In the buried bottle and foliar spray experiments, it was found that buried bottle with foliar spray of Fe-EDTA-Urea significantly corrected apple yellowing by increasing the relative chlorophyll content, improving photosynthetic performance, and increasing iron utilization (Guo et al., 2018). Among others, inorganic iron fertilizer and ferrous sulfate can also be applied to the soil or iron fertilizer can be injected into the trunk to promote plant growth, such as using trunk injection to apply additional iron fertilizer during the budding, flowering, and fruit growth periods of gray jujube, which can improve the growth and quality of gray jujube to some extent (Song et al., 2013); using trunk injection to supplement tree iron nutrition during the fertility period of fruits can promote the growth and expansion of fruit, improve the yield of fruit trees and promote fruit quality (Ahmadi et al., 2023). Although the duration of iron fertilization application is short and the cost is relatively high, it is fast and effective.

### 3.2 Application of soil organic iron fertilizer/soil conditioner

Long-term application of inorganic iron fertilizer will not have an obvious effect on the treatment of plant yellowing disease, and ferrous sulfate is easy to form insoluble high iron substances in the soil, which eventually leads to the deterioration of the plant root environment. In contrast, soil organic fertilizers not only have high iron content, stable performance, and are mostly water-soluble, but also can increase the organic matter content in the soil, promote the release of soil nutrients, maintain the effectiveness of soil nutrients, improve the iron transfer capacity in plants and promote iron uptake by plants (Mohammad et al., 2018). For example, EDTA, DTPA (diacetyltriaminepentaacetic acid), HEDTA (hydroxyethyl ethylenediaminetetraacetic acid), and poultry manure can effectively increase the effective iron content in the soil, improve the symptoms of iron deficiency in plants, increase fruit weight and yield, and improve fruit quality, with obvious results in balsam pear (Ana et al., 2004).

The application of soil amendments to the soil can also improve the nutrient structure of the soil, such as applying acidifiers to the soil to lower the soil pH, thus increasing the effective iron content in the soil and thus improving plant yellowing disease (Dvořáčková et al., 2022); Si can increase the concentration of light and pigments, reduce lipid peroxidation, improve the efficiency of iron transport and utilization, and greater dry weight accumulation, and thus can be applied to reduce plant Fe deficiency symptoms by Si application (Gelza et al., 2020).

### 3.3 Application of microbial fertilizer

Direct application of inorganic iron fertilizer and chelate iron fertilizer has been widely used in production, but there are some problems such as high cost and easy degradation after application in soil (Nosheen et al., 2021). Microbial fertilizers are promising alternatives to chemical fertilizers and are becoming increasingly important in achieving sustainable agriculture. Biofertilization, also known as biofertilization and fungal fertilizer, can increase the supply of plant nutrients, promote plant growth, and improve agricultural products and agro-ecological environment through the life activities of microorganisms contained in it (Nosheen et al., 2021). Biofertilizer are microbial preparations composed of PGPR (plant growth-promoting rhizobacteria) that can directly or indirectly promote plant growth by dissolving soil nutrients and producing plant growth stimulating hormones and ferritic metabolites (Aloo et al., 2022). When applied in combination with Bacillus or Bacillus like species, PGPR plays an important role in improving the growth, yield and quality of maize (Ahmad et al., 2023). Other studies have shown that plant growth promoting microorganisms produce various biomolecules, enhance the content of all the macronutrient synthesis micronutrients available in EFB (empty fruit bunches) biomass, transform them from the form that cannot be obtained by plants to the form that can be absorbed, and regulate the availability of iron in plants (Mahmud and Chong, 2021). The ground balloon mold can enhance the activity of trivalent iron reductase in the root system of Hovenia solids seedlings under alkaline conditions and reduce the production of its iron deficiency yellowing disease Fe-SPBs (siderophore producing bacterial strains) and soil fertilizer, can promote growth of peanut total iron absorption potential (Wang and Xia, 2009; Sarwar et al., 2022). Kumar et al. (2021) revealed the promise of rhizobium and plant growth-promoting rhizosphere bacterial aggregates for increasing biological yield and iron content of lentil seeds.

### 3.4 Crop intercropping

Intercropping is a common agricultural practice improving acquisition of nutrients including iron (Fe) (Noushin et al., 2020). There are generally two ways to change the iron nutrition of crops through intercropping. The first is to change the soil pH. For example, peanut-sesame intercropping can reduce the rhizosphere pH of peanut and increase the available iron content in soil, thus increasing the active iron content in various organs above ground of peanut and effectively improving the iron deficiency yellowing phenomenon of peanut (Wang et al., 2019). For leguminous plants, the improvement of iron nutrition can improve the formation of nodules and enhance the nitrogen fixation ability of roots (Ding et et al., 2009). The second is to secrete chelates to increase the absorption of iron in the soil. For example, grass can absorb iron from calcareous soil more effectively than dicotyledons, Iron deficiency in olives can be alleviated when they are intercropping with purple false brome and barley (Cañasveras et al., 2014). In maize-peanut intercropping, the insoluble iron in soil was chelated by wheat root acids secreted by maize roots, which improved the absorption and utilization of iron by peanuts and alleviated its yellowing (Zuo and Zhang, 2009; Dai et al., 2019).

## 4 Prospection

Iron plays an important role not only in human life but also in the growth and development of plants. Research on iron nutrition in plants has made great progress, and the molecular mechanisms of iron regulation in plants have also made great progress. In previous studies, the mechanisms of iron uptake in plants were divided into 2 types of iron uptake mechanisms dicotyledons and non-grass plants (Mechanism I) and grass plants (Mechanism II). However, plants do not live independently; therefore, the following studies should be enhanced in subsequent research.

Research on the genetic mechanism of plant tolerance to iron deficiency stress, continue to screen relevant regulatory genes, enrich the study of different genes on plant adaptation to iron deficiency, improve the genetic framework of plant response to iron deficiency, and actively apply biological factors such as plant ferritin and nicotinamide synthase to enhance plant iron adaptation to the environment.

Strengthen research on non-adaptive mechanisms. Plants absorb iron from soil by interacting with microorganisms. When the content of reducing iron in soil is insufficient, plants will sense the iron deficiency signal and transmit it to rhizosphere microorganisms, inducing them to secrete iron carriers for plant utilization and absorption. With the gradual deepening of research on microbial nutrition, the role of microorganisms in fertilizers has received increasing attention. The use of biological iron fertilizers can not only improve their fertilizer utilization rate, but also achieve the goal of environmental protection, ensuring the normal growth of plants while reducing fertilizer input, and protecting the environment. Currently, the production and application of microbial iron fertilizer is still in its infancy, and most studies on the amount of microbial fertilizer focus on whether it will have an impact on the environment. Subsequent research can focus on screening suitable microorganisms, such as Bacillus subtilis and soybean rhizobia, to explore the synthesis or degradation of specific molecules involved in plant iron absorption physiology (such as cellulose, hemicellulose, humic acid, and plant hormones), and to improve the mechanism by which plants absorb iron through the mechanism of microbial plant interaction through changes in microbial genes that affect plant traits, Provide a theoretical basis for production and application.

Strengthen the research application of iron deficiency mechanism in fruit arboriculture. As far as the existing research finds, the research on iron deficiency mechanism is mainly concentrated in legumes, rice, and other crops, and relatively little research is done in fruit trees. Therefore, it is important to strengthen the research on fruit trees to reduce the yield decline caused by soil iron deficiency, improve fruit quality, and enhance economic efficiency.

Continued screening of genotypic varieties against iron deficiency stress and screening of genotypic plants with high resistance to iron deficiency has become a fundamental way to overcome plant tolerance to iron deficiency stress.

## Author contributions

XN and ML are the main author of the review, completing the search, collection, analysis, and writing of the first draft of relevant literature; XW and ZG are the creators and principals of the article; GH and JM participated in the writing and revision of the article. All the authors contributed to the article and approved the submitted version.
